# Supplementary material for: De-Escalation of Anti-Tumor Necrosis Factor Alpha Agents and Reduction in Adverse Effects: A Systematic Review
Source: Biomedicines. 2022 Apr 29;10(5):1034. doi: 10.3390/biomedicines10051034 (PMC9139158; doi:10.3390/biomedicines10051034)
Supplement: Supplementary file 1 [file biomedicines-10-01034-s001.zip › biomedicines-1675878-supplementary.pdf]

Supplementary Materials:

Title: De-escalation strategies of anti-tumor necrosis factor agents and reduction of adverse effects: a systematic review

Table S1: PRISMA 2020 checklist;

Table S2: Search strategies;

Table S3: Detailed description of standard treatment and de-escalation method used in the included studies

Table S1: PRISMA 2020 checklist

Section and Topic	Item #	Checklist item	Location where item is reported
**TITLE**			
Title	1	Identify the report as a systematic review.<br><br>*"De-escalation strategies of anti-tumor necrosis factor agents and reduction of adverse effects: a systematic review"*	*Title page*
**ABSTRACT**			
Abstract	2	See the PRISMA 2020 for Abstracts checklist.	*Abstract*
**INTRODUCTION**			
Rationale	3	Describe the rationale for the review in the context of existing knowledge.<br><br>*"The downside of long-term exposure to anti-TNF agents is its association with adverse effect such as immune-mediated cutaneous reactions and susceptibility to infections.(…)"*<br><br>*"Multiple systematic reviews and meta-analyses of de-escalation studies demonstrated little or no increase in disease activity compared with standard dosing.(…)"*<br><br>*"It is unknown whether de-escalation reduces adverse effects."*	*Introduction,*<br><br>*Paragraph 2*<br><br>*Paragraph 4*<br><br>*Last paragraph*
Objectives	4	Provide an explicit statement of the objective(s) or question(s) the review addresses.<br><br>*"To evaluate the incidence of infections and skin manifestations after anti-TNF de-escalation, irrespective of the underlying inflammatory condition. The outcomes of interest included disappearance as well as reduction of infections and skin manifestations and the rate of occurrence of new infections and skin manifestations."*	*Introduction, last paragraph*
**METHODS**			
Eligibility criteria	5	Specify the inclusion and exclusion criteria for the review and how studies were grouped for the syntheses.<br><br>*"To be included in this systematic review, studies had to meet the following eligibility criteria: Full-text articles of randomized controlled trials (RCTs) or observational cohort or case-control studies involving patients treated with standard-dosed anti-TNF-agents (adalimumab, infliximab, certolizumab pegol, golimumab or etanercept), undergoing anti-TNF de-escalation (dose reduction or interval lengthening), containing information about the rate of disappearance and/or reduction of infections and/or skin manifestations and/or the rate of occurrence of new infections and/or skin manifestations. (…) Studies in which de-escalation resulted in discontinuation without separate information about adverse effects during the de-escalation phase were excluded. Case reports, case series and studies with a cross-sectional design were also excluded."*	*Methods – Study selection, first paragraph*
Information sources	6	Specify all databases, registers, websites, organisations, reference lists and other sources searched or consulted to identify studies. Specify the date when each source was last searched or consulted.<br><br>*"We searched MEDLINE (through PubMed), EMBASE and the Cochrane Library from inception to 14 January 2022. (…) All searches were carried out on 14 January 2022. (…) In addition, we hand-searched references of relevant publications to identify additional studies that were missed in the database searches."*	*Methods – Data sources and searches*
Search strategy	7	Present the full search strategies for all databases, registers and websites, including any filters and limits used.<br><br>*"The search strategies were developed in collaboration with a medical information specialist and consisted of a Boolean association of*	*Methods – Data sources and*

Section and Topic	Item #	Checklist item	Location where item is reported
		keywords, combining keywords for anti-TNF agents and de-escalation. The search strategies for each of the electronic databases are shown in table S2."	*searches; Table S2*
Selection process	8	Specify the methods used to decide whether a study met the inclusion criteria of the review, including how many reviewers screened each record and each report retrieved, whether they worked independently, and if applicable, details of automation tools used in the process.<br><br>*"The search results were imported into EndNote X9 for de-duplication,[21] and subsequently imported into Rayyan, an online tool for systematic reviews.[22] Two reviewers (MB, PvR) independently reviewed titles and abstracts for eligibility. In case eligibility was unclear based on title and abstract, the record was included for full-text assessment. Disagreements were solved by referral to a third reviewer (WL). Next, full-text articles were screened independently by the same two reviewers. Again, disagreements were solved by referral to the third reviewer."*	*Methods - Study selection, paragraph 2*
Data collection process	9	Specify the methods used to collect data from reports, including how many reviewers collected data from each report, whether they worked independently, any processes for obtaining or confirming data from study investigators, and if applicable, details of automation tools used in the process.<br><br>*"Data was extracted by one reviewer (MB) with confirmation by another reviewer (PvR). (…)Missing data was requested from study authors via email."*	*Methods - Data extraction and quality assessment, first paragraph*
Data items	10a	List and define all outcomes for which data were sought. Specify whether all results that were compatible with each outcome domain in each study were sought (e.g. for all measures, time points, analyses), and if not, the methods used to decide which results to collect.<br><br>*"The following characteristics were extracted from each selected study: (…)incidence/prevalence and/or disappearance/reduction of infections and/or skin manifestation at baseline and at each reported time point, type and severity of the adverse reactions. "*	*Methods - Data extraction and quality assessment, first paragraph*
	10b	List and define all other variables for which data were sought (e.g. participant and intervention characteristics, funding sources). Describe any assumptions made about any missing or unclear information.<br><br>*"The following characteristics were extracted from each selected study: first author, year of publication, report title, name of study, corresponding author's contact information, country of origin, publication type, study design, in- and exclusion criteria, allocation method, anti-TNF dosing (standard dosing and de-escalation method), start date, end date, duration of participation, sample size, baseline imbalances, withdrawals and exclusions, patient characteristics at baseline (age, sex, diagnosis, anti-TNF agent(s) used, co-treatment) (…)Missing data that could not be obtained was presented as 'not provided' and was not included in the syntheses."*	*Methods - Data extraction and quality assessment, first paragraph*
Study risk of bias assessment	11	Specify the methods used to assess risk of bias in the included studies, including details of the tool(s) used, how many reviewers assessed each study and whether they worked independently, and if applicable, details of automation tools used in the process.<br><br>*"The risk of bias for the outcome of interest of of RCTs was assessed with the revised Cochrane risk-of bias tool (RoB 2).[23] The risk of bias was scored by two reviewers (MB, PvR) as 'low', 'some concerns' or 'high' for each domain individually and overall. These results were displayed in a figure. The risk of bias of non-RCTs was assessed with the Newcastle-Ottawa quality assessment Scale (NOS) and converted to the Agency for Healthcare Research and Quality standards ('good', 'fair' or 'poor') and displayed in a table.[24, 25]"*	*Methods - Data extraction and quality assessment, last paragraph*
Effect measures	12	Specify for each outcome the effect measure(s) (e.g. risk ratio, mean difference) used in the synthesis or presentation of results.<br><br>*"The occurrence of infections and skin manifestations was presented as the proportion of patients with at least one event for the standard*	*Methods - Data analysis and*

Section and Topic	Item #	Checklist item	Location where item is reported
		treatment and the de-escalation group for each study individually. 95% confidence intervals (CIs) were calculated for these proportions using the Wilson method. Difference in these proportions were considered to be statistically significant if there was no overlap in the 95% confidence intervals of the two groups or if the p-value was below 0.05, if provided. A relative difference of ≥25% was considered numerically different."	*synthesis of results, first paragraph*
Synthesis methods	13a	Describe the processes used to decide which studies were eligible for each synthesis (e.g. tabulating the study intervention characteristics and comparing against the planned groups for each synthesis (item #5)).<br><br>*"The occurrence of infections and skin manifestations was presented as the proportion of patients with at least one event for the standard treatment and the de-escalation group for each study individually. 95% confidence intervals (CIs) were calculated for these proportions using the Wilson method. Difference in these proportions were considered to be statistically significant if there was no overlap in the 95% confidence intervals of the two groups or if the p-value was below 0.05, if provided. A relative difference of ≥25% was considered numerically different."*	*Methods - Data analysis and synthesis of results, first paragraph*
	13b	Describe any methods required to prepare the data for presentation or synthesis, such as handling of missing summary statistics, or data conversions.<br><br>*"Missing data was requested from study authors via email. Missing data that could not be obtained was presented as 'not provided' and was not included in the syntheses."*	*Methods - Data extraction and quality assessment, first paragraph*
	13c	Describe any methods used to tabulate or visually display results of individual studies and syntheses.<br><br>*"The risk of bias of RCTs was (…) displayed in a figure. The risk of bias of non-RCTs was (…) displayed in a table."*<br><br>*"(…) main results of included studies were presented in a table."*	*Methods - Data extraction and quality assessment, last paragraph*<br><br>*Methods - Data analysis and synthesis of results, first paragraph*
	13d	Describe any methods used to synthesize results and provide a rationale for the choice(s). If meta-analysis was performed, describe the model(s), method(s) to identify the presence and extent of statistical heterogeneity, and software package(s) used.<br><br>*"Results were summarized narratively and characteristics and main results of included studies were presented in a table."*	*Methods - Data analysis and synthesis of results, first paragraph*
	13e	Describe any methods used to explore possible causes of heterogeneity among study results (e.g. subgroup analysis, meta-regression).	*N/A*
	13f	Describe any sensitivity analyses conducted to assess robustness of the synthesized results.	*N/A*
Reporting bias	14	Describe any methods used to assess risk of bias due to missing results in a synthesis (arising from reporting biases).	*N/A*

Section and Topic	Item #	Checklist item	Location where item is reported
assessment			
Certainty assessment	15	Describe any methods used to assess certainty (or confidence) in the body of evidence for an outcome.	*N/A*
**RESULTS**			
Study selection	16a	Describe the results of the search and selection process, from the number of records identified in the search to the number of studies included in the review, ideally using a flow diagram.<br><br>*"A total of 2280 articles were identified of which 128 were retrieved for full text review. Of these, 108 were excluded as they did not report the outcome of interest (Fig 1)."*	*Results, first paragraph; Fig 1*
	16b	Cite studies that might appear to meet the inclusion criteria, but which were excluded, and explain why they were excluded.<br><br>*"Reports excluded:*<br>*Wrong study design (n = 1)*<br>*No or insufficient information about outcome of interest (n = 106)*<br>*Subgroup of included study 3 (n = 1)"*	*Fig 1*
Study characteristics	17	Cite each included study and present its characteristics.<br><br>*"Table 1 lists the characteristics of 20 studies that compared any form of anti-TNF dose de-escalation with standard dosing.[27-46]"*	*Results, first paragraph; Table 1*
Risk of bias in studies	18	Present assessments of risk of bias for each included study.<br><br>*"Of these, 14 were RCTs (2197 patients; eight trials with low risk of bias or some concerns, Fig 2).[27-39] Four of six non-RCTs (509 patients) were of good methodological quality (Table 2).[41-46]"*	*Results, first paragraph; Fig 2; Table 2*
Results of individual studies	19	For all outcomes, present, for each study: (a) summary statistics for each group (where appropriate) and (b) an effect estimate and its precision (e.g. confidence/credible interval), ideally using structured tables or plots.<br><br>*"Main results adverse effects (n of patients with ≥1 event (%, 95% CI), unless otherwise specified)"*	*Table 1*
Results of syntheses	20a	For each synthesis, briefly summarise the characteristics and risk of bias among contributing studies.<br><br>***Occurrence of new infections***<br>*"Seventeen of twenty articles reported on the occurrence of new infections during the study observation period.[27-34, 36-43, 45, 47]"*<br><br>*And onwards: subheadings 'occurrence of new infections', 'Disappearance of infections', 'Occurrence of new skin manifestations' and 'Disappearance of skin manifestations'.*	*Results – (see left column)*
	20b	Present results of all statistical syntheses conducted. If meta-analysis was done, present for each the summary estimate and its precision (e.g. confidence/credible interval) and measures of statistical heterogeneity. If comparing groups, describe the direction of the effect.	*N/A*
	20c	Present results of all investigations of possible causes of heterogeneity among study results.	*N/A*

Section and Topic	Item #	Checklist item	Location where item is reported
	20d	Present results of all sensitivity analyses conducted to assess the robustness of the synthesized results.	*N/A*
Reporting biases	21	Present assessments of risk of bias due to missing results (arising from reporting biases) for each synthesis assessed.	*N/A*
Certainty of evidence	22	Present assessments of certainty (or confidence) in the body of evidence for each outcome assessed.	*N/A*
**DISCUSSION**			
Discussion	23a	Provide a general interpretation of the results in the context of other evidence.<br><br>*"Our findings are consistent with a meta-analysis performed by Vinson et al. They evaluated the incidence of serious infections and adverse events of specific interest in patients with RA or axial spondyloarthritis. Thirteen studies were included in the meta-analysis, seven of which are also included in our systematic review. De-escalation of the biological disease-modifying anti-rheumatic drug (predominantly anti-TNF agents) or JAK inhibitor was not different from continuation of the initial regimen with respect to the incidence of serious infections (risk difference 0.01, 95% CI -0.00-0.02, p=0.13, I2=0%). Neither non-serious infections nor skin manifestations were taken into consideration.[48]*<br>*Likewise, a Cochrane systematic review on down-titration and discontinuation of anti-TNF agents in patients with RA did also not report on the occurrence of infections or skin manifestations. Based on five studies, four of which are also included in our systematic review, the authors concluded that de-escalation has little to no effect on serious adverse effects, but the evidence was also very uncertain.[13]"*	*Discussion, paragraph 3+4*
	23b	Discuss any limitations of the evidence included in the review.<br><br>*"Although in our systematic review eight RCTs and four non-RCTs were of good overall methodological quality, measurement of the outcome of interest was problematic. None of the studies defined adverse effects as their primary outcome. Instead, data about infections or skin manifestations were at best presented as part of the obligatory reporting of adverse events. As a consequence, most studies were not powered to detect potential differences in the occurrence of adverse events. Additionally, clear descriptions of the definitions and methods of measurement were lacking.*<br>*In most studies the occurrence of new infections was expressed as incidences. This may have resulted in an underestimation of the effect of anti-TNF de-escalation on adverse effects. For instance, if a patient in the standard treatment group had 10 infections during follow-up and another patient in the de-escalation group had only 1, this would not result in a difference in incidences, whereas the use of event rates would have provided a more reliable estimate."*	*Discussion, paragraph 5+6*
	23c	Discuss any limitations of the review processes used.<br><br>*"We only included publications that reported on infections, skin manifestations or both. Because of the obligatory reporting of adverse events, it is likely that this data was also available for de-escalation studies that did not provide this information in their publication. In fact, 106 studies were excluded during the full-text selection because they contained no or insufficient information about the adverse events of interest. This may have caused selection bias, but obtaining these missing data from such a large number of publications would not be feasible."*	*Discussion, paragraph 8*
	23d	Discuss implications of the results for practice, policy, and future research.<br><br>*"There are multiple reasons why both patients and healthcare professionals may wish to de-escalate anti-TNF therapy once remission has been achieved. These reasons include a reduction in the number of hospital visits, the number of needle pricks and costs. Reduction of anti-TNF associated adverse effects is also often mentioned as a reason, but we cannot confirm the validity of this approach. We suggest not to de-escalate standard-dosed anti-TNF medication solely for this reason. This advice does not apply to patients treated with a shorter dosing interval, a higher dose than standard, or both."*	*Discussion, paragraph 2*<br><br>*Discussion,*

Section and Topic	Item #	Checklist item	Location where item is reported
		"Further research is necessary for better quality data on the possible beneficial effect of anti-TNF de-escalation on anti-TNF associated adverse effects. This is of particular importance for patients with IBD and psoriasis and the anti-TNF agents infliximab, certolizumab and golimumab, who were underrepresented in the current review. To better address the question whether de-escalation reduces adverse effects, future studies should scrupulously register the adverse effects of interest, for instance by sending out questionnaires specifically designed for this purpose.[48]"	*paragraph 9*
**OTHER INFORMATION**			
Registration and protocol	24a	Provide registration information for the review, including register name and registration number, or state that the review was not registered.<br><br>*"The study protocol was registered on PROSPERO (CRD42021252977), prior to the literature search."*<br><br>*https://www.crd.york.ac.uk/prospero/display_record.php?RecordID=252977*	*Methods, first paragraph*
	24b	Indicate where the review protocol can be accessed, or state that a protocol was not prepared.<br><br>*"The study protocol was registered on PROSPERO (CRD42021252977), prior to the literature search."*<br><br>*https://www.crd.york.ac.uk/prospero/display_record.php?RecordID=252977*	*Methods, first paragraph*
	24c	Describe and explain any amendments to information provided at registration or in the protocol.	*N/A*
Support	25	Describe sources of financial or non-financial support for the review, and the role of the funders or sponsors in the review.<br><br>*"Funding: This research received no external funding."*	*Funding*
Competing interests	26	Declare any competing interests of review authors.<br><br>*"Conflicts of Interest: The authors declare no conflict of interest."*	*Conflicts of Interest*
Availability of data, code and other materials	27	Report which of the following are publicly available and where they can be found: template data collection forms; data extracted from included studies; data used for all analyses; analytic code; any other materials used in the review.<br><br>*"Data Availability Statement: Not applicable."*	*Data Availability Statement*

Table S2: Search strategies

Database	Search strategy
MEDLINE	("Tumor Necrosis Factor Inhibitors"[Mesh] OR "Tumor Necrosis Factor-alpha/antagonists and inhibitors"[Mesh] OR "Tumor Necrosis Factor Inhibitors" [Pharmacological Action] OR "Infliximab"[Mesh] OR "Adalimumab"[Mesh] OR "Certolizumab Pegol"[Mesh] OR "Etanercept"[Mesh] OR “anti-TNF*”[tiab] OR “Anti-Tumour Necrosis Factor*”[tiab] OR “Anti-Tumor Necrosis Factor*”[tiab] OR “Tumor Necrosis Factor inhibitor*”[tiab] OR “Tumour Necrosis Factor inhibitor*”[tiab] OR “TNF inhibitor*”[tiab] OR “TNF-a inhibitor*”[tiab] OR “TNF-alpha inhibitor*”[tiab] OR “TNFa inhibitor*”[tiab] OR “TNFalpha inhibitor*”[tiab] OR “Tumor Necrosis Factor Block*”[tiab] OR “Tumour Necrosis Factor Block*”[tiab] OR “TNF block*”[tiab] OR “TNF-a block*”[tiab] OR “Tumor Necrosis factor-a block*”[tiab] OR “Tumor Necrosis Factor Antagonist*”[tiab] OR “Tumour Necrosis Factor Antagonist*”[tiab] OR “TNF Antagonist*”[tiab] OR “TNF-a Antagonist*”[tiab] OR “Infliximab”[tiab] OR “Adalimumab”[tiab] OR “Certolizumab”[tiab] OR “Etanercept”[tiab] OR “Golimumab”[tiab])<br>AND<br>(“De-escalat*”[tiab] OR “Deescalat*”[tiab] OR “Dose reduction”[tiab] OR “Reduced dose*”[tiab] OR “Interval lengthening”[tiab] OR “Lengthened interval”[tiab] OR (Lengthened[tiab] AND “dosing interval”[tiab]) OR “Taper*”[tiab] OR “Down-titrat*”[tiab] OR “Downtitrat*”[tiab] OR “half dose”[tiab] OR (“spaced”[tiab] AND “dose”[tiab]))
EMBASE	(‘tumor necrosis factor inhibitor’/exp OR ‘certolizumab pegol’/exp OR (((anti OR inhibit* OR blocker* OR blocking OR antagonis*) NEAR/2 (tnf OR “tumour necrosis factor*” OR “tumor necrosis factor*”)) OR “Infliximab” OR “Adalimumab” OR “Certolizumab” OR “Etanercept” OR “Golimumab”):ab,ti)<br>AND<br>(“De-escalat*” OR “Deescalat*” OR “Dose reduction” OR “Reduced dose*” OR “Interval lengthening” OR “Lengthened interval” OR (Lengthened AND “dosing interval”) OR “Taper*” OR “Down-titrat*” OR “Downtitrat*” OR “half dose” OR (“spaced” AND “dose”)):ab,ti<br>NOT<br>[conference abstract]/lim
Cochrane Library	([mh "Tumor Necrosis Factor Inhibitors"] OR [mh "Tumor Necrosis Factor-alpha"] OR [mh infliximab] OR [mh adalimumab] OR [mh "certolizumab pegol"] OR [mh etanercept] OR (((anti OR inhibit* OR blocker* OR blocking OR antagonis*) NEAR/2 (tnf OR tumour-necrosis-factor* OR tumor-necrosis-factor*)) OR “Infliximab” OR “Adalimumab” OR “Certolizumab” OR “Etanercept” OR “Golimumab”):ab,ti)<br>AND<br>((De-escalat* OR Deescalat* OR “Dose reduction” OR Reduced-dose* OR “Interval lengthening” OR “Lengthened interval” OR (Lengthened AND “dosing interval”) OR Taper* OR Down-titrat* OR Downtitrat* OR “half dose” OR (“spaced” AND “dose”)):ab,ti)

Table S3: Detailed description of standard treatment and de-escalation method used in the included studies

Study	Standard treatment	De-escalation strategy
Papp (Br J Dermatol 2005)	Etanercept 50 mg twice weekly	Etanercept 25 mg twice weekly
Smolen (Lancet 2013)	Etanercept 50 mg twice weekly	Etanercept 25 mg twice weekly
Cantini (Biologics 2013)	Etanercept 50 mg weekly	Etanercept 50 mg every other week
Emery (N Engl J Med 2014)	Etanercept 50 mg weekly	Etanercept 25 mg weekly
Yates (J Rheum 2015)	Etanercept 50 mg weekly	Etanercept 25 mg weekly
Raffeiner (Clin Exp Rheumatol 2015)	Etanercept 25 mg twice weekly	Etanercept 25 mg weekly
Li (Int J Immunopathol Pharmacol 2016)	Etanercept 25 mg twice weekly	Etanercept 25 mg weekly
Weinblatt (Arthritis Rheumatol 2017)	Certolizumab pegol 200 mg every other week	Certolizumab pegol 200 mg every 4 weeks
Ibrahim (Rheumatology (Oxford) 2017)	Etanercept 50 mg weekly; Adalimumab 40 mg every other week	Step-wise tapering to 33% or 66% of the initial dose: Etanercept: 50 mg twice every 3 weeks (50-50-0 mg, 33% tapering) or 50 mg every 3 weeks (66% tapering); Adalimumab: 40 mg twice every 3 weeks (40-40-0 mg, 33% tapering) or 40 mg every 3 weeks (66% tapering)
Gratacós (Arthritis Res Ther 2019)	Adalimumab 40 mg every other week; Etanercept 25 mg every 3 days or 50 mg every 7 days; Golimumab 50 mg every 4 weeks; Infliximab 5 mg/kg every 6–8 weeks	Adalimumab 40 mg every 3 weeks; Etanercept 50 mg every 10 days; Golimumab 50 mg every 6 weeks; Infliximab 3 mg/kg every 8 weeks
Atalay (JAMA Dermatol 2020)	Adalimumab every other week; Etanercept every week	Adalimumab: step 1: every 3 weeks; step 2: every 4 weeks; Etanercept: step 1: every 10 days; step 2: every other week Step 2 after 3 months depending on disease activity.
Landewé (Ann Rheum Dis 2020)	Certolizumab pegol 200 mg every other week	Certolizumab pegol 200 mg every 4 weeks
Emery (Ann Rheum Dis 2020)	Adalimumab 40 mg every other week	Adalimumab 40 mg every 3 weeks
Bertrand (Scand J Rheumatol 2021)	Etanercept 50 mg every week	Etanercept 50 mg every other week
Park (Clin Exp Rheumatol 2016)	Etanercept 25 mg twice weekly or 50 mg weekly	Step 1: Etanercept 25 mg weekly or 50 mg every other week. Further reduction considered if clinical remission continued for the following 6-12 months (not specified)
Závada (Ann Rheum Dis 2016)	Adalimumab: not specified; Etanercept: 50 mg weekly; Infliximab: not specified	80.7% underwent interval lengthening (most often with etanercept); 14.0% underwent dose-reduction (most often with infliximab); 5.3% underwent a combination of interval lengthening and dose-reduction
Li (Arch Med Sci 2019)	Etanercept 50 mg weekly	Etanercept 25 mg weekly
Pouillon (Dig Liver Dis 2019)	Adalimumab 40 mg every other week	Adalimumab 40 mg every 3 weeks
Atalay (J Dermatolog Treat 2021)	Adalimumab every other week; Etanercept every week	Adalimumab: step 1: every 3 weeks; step 2: every 4 weeks; Etanercept: step 1: every 10 days; step 2: every other week Step 2 after 3 months depending on disease activity.

van Steenbergen (Aliment Pharmacol Ther 2017)	Adalimumab 40 mg every other week	Adalimumab 40 mg every 3 weeks
